# Prevalence and Characteristics of Thyroid Abnormalities and Its Association with Anemia in ASIR Region of Saudi Arabia: A Cross-Sectional Study

**DOI:** 10.3390/clinpract11030065

**Published:** 2021-08-06

**Authors:** Saif Aboud M. Alqahtani

**Affiliations:** Internal Medicine Department, College of Medicine, King Khalid University, P.O. Box 641, Abha 61421, Saudi Arabia; saifalqahtani544@gmail.com or saif@kku.edu.sa; Tel.: +966-50575-4832

**Keywords:** thyroid dysfunction, subclinical hypothyroidism, hematological profile, anemia, microcytic hypochromic anemia, normocytic normochromic anemia

## Abstract

The thyroid gland plays a significant role in the metabolism and proliferation of blood cells; hematological disorders are frequently observed in patients with thyroid disorders, and the most frequent problem is anemia. The main objective of this research work is to evaluate the prevalence and types of thyroid dysfunction and their association with anemia in different gender stratified by age in the Asir region of Saudi Arabia. Four years of data from July 2016 to July 2020 for 9992 study subjects were collected. Statistical analysis was performed based on thyroid disorder and anemia stratified by gender and age subgroup. The mean age of the study subject was 43.4 ± 15.8 years, and females constituted 61.7% of cases. Thyroid dysfunction was observed in 49.76% (4973), and subclinical hypothyroidism was the most prevalent type (3922/9992), followed by primary hypothyroidism (530/9992). Females have a significantly higher overall prevalence of thyroid dysfunction than males (*p* < 0.05). Anemia was detected in 1344 females and 465 males with a thyroid disorder, and also, the prevalence was significantly higher (*p* < 0.05), compared to the normal thyroid group. Thyroid disorders are a common problem in our population, more prevalent in females than males, with the peak age of above 30 years, and are associated with an increased prevalence of anemia.

## 1. Introduction

The thyroid gland is one of the most vital organs that play a critical role in the normal growth, differentiation, metabolism, and physiological functioning of the human body. Thyroid dysfunction is one of the most common problems in clinical practice and has become more prevalent throughout the world in recent decades; therefore, its associated risk factors have received much attention [1,2]. It can be caused by over- or under-secretion of thyroid hormones and abnormalities in thyroid hormone receptors. The prevalence of thyroid disorders depends on sex, age, and geographical location, and dietary variation of iodine intake [3,4]. It is easily identifiable treatable, but it can have severe consequences if left undiagnosed or untreated [5]. Thyroid dysfunction significantly impacts health outcomes, including cardiovascular, metabolic dysfunction, metabolic disorders, mental, and bone health [5,6,7].

The thyroid gland is one of the most important endocrine glands in the human body that plays a critical role in the body, including metabolism and erythropoiesis by induction of erythropoietin secretion and proliferation of erythroid progenitors [8,9,10]. Thyroid disorder can lead to a wide range of symptoms, including hypoplasia of erythroid cells in the bone marrow or proliferation of immature erythroid progenitor cells (due to hypothyroidism), or hyperplasia (due to hyperthyroidism) [10,11]. In general, thyroid dysfunction can lead to different effects on blood cells and anemia of multifarious severity and types [9,10,12,13,14,15]. The prevalence of thyroid dysfunction and hematological abnormalities is well known [16,17]. Decreased number of red blood cells (RBC) was observed in patients with thyroidectomy [17], and anemia (microcytic and normocytic anemia) has been reported in almost 25–55% of the patient with thyroid hypothyroidism [9,13,18,19]. Similarly, earlier reports demonstrate the association of Graves’s disease with anemia [17,20,21].

Females are 10 times more prone to develop thyroid dysfunction and associated anemia and other diseases than males [18,22,23]. Little information is available about the prevalence of thyroid disorder and associated anemia in the Kingdom of Saudi Arabia (KSA). Minimal studies have been conducted in a primary setting, whereas none are carried out among the general population. Therefore, this study aimed to assess the prevalence and types of thyroid disorder and their relationship with anemia and types of anemia stratified by gender and age in the Asir region of KSA.

## 2. Materials and Methods

### 2.1. Study Setting

The Asir region is located in the southwest region of Saudi Arabia. It has an area of 76,693 square kilometers that extends from the high Asir Mountains, almost 3200 m above sea level, down to the Red Sea. According to the statistics for 2017, its population is around 2.212 million. Asir receives up to 500 mm of rain annually, has one of the kingdom’s wetter and more temperate climates, and is an important agricultural region. Abha and Khamis-Mushayt are the main towns of this region. 

### 2.2. Precipitant and Data Collection

This cross-sectional study was conducted between July 2016 to July 2020 in the Asir region of Saudi Arabia. A total of 9992 lab data from different commercial clinical laboratories located at various locations in Abha and Khamis-Mushayt were retrieved. Selected participants were either customers who came for a regular health check-up or follow-up of chronic health conditions. In the lab, after an 8 h of overnight fasting, 5 mL blood samples were collected from all the participants at the clinical laboratory. From the collected blood sample, 2.5 mL were used for measurement of red blood cell (RBC) count, red cell distribution width (RDW), hematocrit (HCT), hemoglobin (Hb), mean corpuscular volume (MCV), mean corpuscular hemoglobin (MCH), and mean corpuscular concentration (MCHC) using hematology analyzer (Abbott, Inc., Wiesbaden, Germany). The remaining 2.5 mL of the whole blood were centrifuged a 2000× *g* for 25 min, and serum samples were used to assess the thyroid-stimulating hormone (TSH) and free tetraiodothyronine (FT4) level by enzyme-linked immunosorbent assay (TSH ELISA kit and FT4 ELISA kit, DIA source, Inc, Ottignies-Louvain-la-Neuve, Belgium).

The reference range for TSH was 0.35 to 4.94 µIU/mL, and the reference range for FT4 was set to 0.7 to 1.48 ng/dL. Study subjects with thyroid dysfunction were divided into four categories [5,24,25,26] as follows: (a) primary hypothyroidism: serum TSH > 4.94 µIU/mL and FT4 < 0.7 ng/dL; (b) primary hyperthyroidism: serum TSH < 0.35 µIU/mL and FT4 > 1.48 ng/dL; (c) subclinical hyperthyroidism: normal FT4 (0.7–1.48 ng/dL) and serum TSH below the lower limit of the reference range (<0.35 µIU/mL); (d) subclinical hypothyroidism: normal FT4 (0.85–1.4 ng/dL) and serum TSH higher than the upper limit of reference range >4.94 µIU/mL. Generally, anemia is defined by low Hb (<12 g/dL in females and <13.5 g/dL in the male), where the subdivision of anemia includes a microcytic hypochromic, which comes with low MCV and low MCH and normocytic normochromic with normal MCV and normal MCH, respectively [12,13,14,15].

### 2.3. Statistical Analysis

Statistical analysis was performed by IBM^®^ SPSS, version 23 (SPSS Inc., Chicago, IL, USA). Person chi-square (χ^2^) and Fisher’s exact test were used for analyzing categorical variables and expressed as frequency and percentage. Kolmogorov–Smirnov test or Shapiro–Wilk test was used the assess the normality of data. Independent-samples T-test was used to analyze two groups for normally distributed data, while Mann–Whitney and Kruskal–Wallis tests were used to compare groups of independent nonparametric variables. Data were expressed as percentages, mean ± standard deviation (SD) for normally distributed continuous data and median ± interquartile range (IQR) for nonnormal continuous data. The linear association between TSH, FT4, and hematological parameters and correlation of these variables with gender and age was assessed using Spearman’s correlation test. The level of statistical significance was set at *p* < 0.05. 

### 2.4. Ethical Approval 

Ethical approval and subject consent waiver were obtained from the research ethics committee at King Khalid University (HAPO-06-B-001) (approval number ECM#2021-4405). 

## 3. Results

### 3.1. General Characteristic of the Study Population

Overall, 9992 study subjects living in the Asir region were involved in this study, with 61.6% (6160) being female and 38.4% (3832) male. All of the study subjects were Saudis. Younger females (31–40 years) formed 31.6% (1949) of the female population, followed by females with age group above 50 (28.7%, 1767) and 41–50 years (20.4%, 1259), respectively. Among the male population, the most representative age group was above 50 years, followed by young males aged 31–40 years (Table 1). 

A significant difference was observed between the mean age of females (42.76 ± 15.64 years) and males (44.50 ± 16.16 years) (*p* < 0.05). No significant difference was observed in terms of TSH, FT4 (*p* > 0.05) between the male and female population (Table 2). However, a statistically significant difference was observed between female and male population for Hb, HCT, MCV, MCH, MCHC, RDW, and RBC levels (*p* < 0.05). General characteristics of the study population stratified by gender are presented in Table 2. 

### 3.2. Thyroid Dysfunction

**Figure 1:**
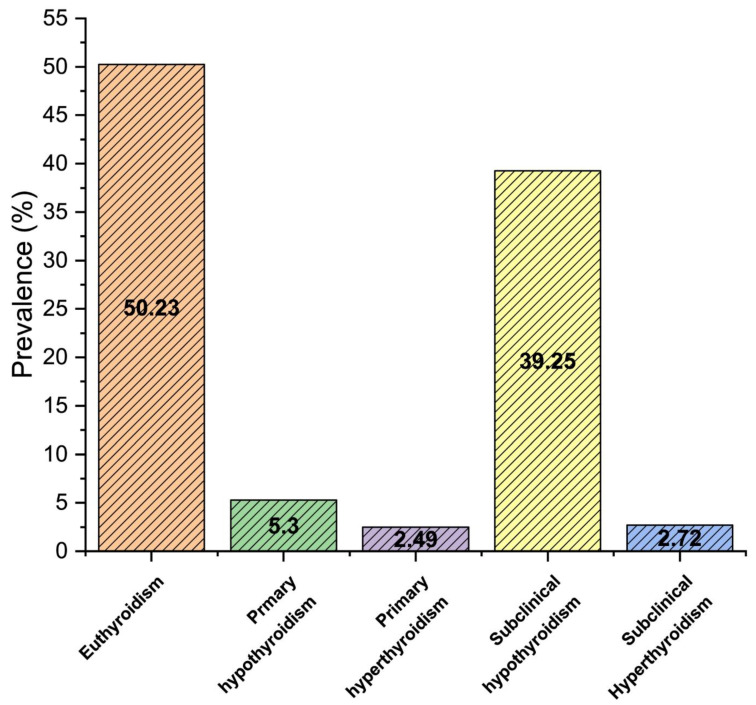
The overall prevalence of thyroid dysfunction in the study population.

Our report demonstrates the prevalence of thyroid dysfunction in 49.8% (4973) population, with 5.3% (530) primary hypothyroidism, 2.5% (249) primary hyperthyroidism, 39.3% (3922) subclinical hypothyroidism, and 2.7% (272) subclinical hyperthyroidism as shown in Figure 1 (*p* < 0.05). The prevalence of thyroid dysfunction was significantly higher in females than males (*p* < 0.05). Furthermore, the prevalence of subclinical hypothyroidism was considerably higher in females (24%), compared to males (15.3%). 

Further detailed analysis of thyroid dysfunction stratified by gender and age group is presented in Table 3. Most age subgroups in females demonstrated a significantly higher prevalence of thyroid dysfunction than the opposite gender of same age sub-groups (*p* < 0.05). Among different age subgroups in females, the prevalence of subclinical hypothyroidism was significantly higher in young females (<20 and 20–30 years) (*p* < 0.05). However, a significantly high prevalence of subclinical hypothyroidism was observed in males below 20 and 31–40 years (*p* < 0.05). The overall prevalence of thyroid dysfunction among the study population stratified by the age subgroup is presented in Table S1.

### 3.3. Thyroid Profile and Anemia

Microcytic hypochromic was observed in 15.8% (1574) female and 6.2% (615) male, and normocytic normochromic anemia in 9.4% (941) female and 3.7% (371) male population, respectively. The prevalence of microcytic hypochromic anemia was significantly higher than normocytic normochromic anemia (*p* < 0.05).

Anemia was observed in 33.71% (1692) study subjects with normal thyroid (euthyroidism), among which there were 14.12% (709) females and 6.35% (319) males with microcytic hypochromic anemia, while 9.20% (462) females and 4.02% (202) males with normocytic normochromic anemia. Moreover, 36.37% (1809) anemia was detected with the thyroid dysfunction group, and the types observed were 17.4% (865) females and 5.95% (296) male with microcytic hypochromic anemia, while 9.63% (479) were females and 3.3% (169) were males with normocytic normochromic anemia. Both types of anemia were significantly more prevalent in the thyroid dysfunction group than the normal thyroid profile (*p* < 0.05). Additionally, the prevalence of microcytic hypochromic anemia was significantly higher (*p* = 0.014) in the thyroid dysfunction group, compared to normocytic normochromic anemia. Prevalence and types of thyroid disorder and anemia stratified by gender are presented in Table 4.

Of the 530 with primary hypothyroidism, anemia was detected in 32.45% (172) cases and types detected as follows: 17.35% (92) female and 2.26% (12) male with microcytic hypochromic anemia, and 10.94% (58) female and 1.88% (10) male with normocytic normochromic anemia. Out of 249 cases with primary hyperthyroidism, anemia was detected in 30.92% (77) cases, and type of anemia was detected as follows: 13.65% (34) female and 2.0% (5) male with microcytic hypochromic anemia, and 14.05% (35) female and 1.20% (3) male with normocytic normochromic anemia. Overall, 17.51% (687) female and 6.88% (270) male with subclinical hypothyroidism (3922) were identified with microcytic hypochromic anemia, and 9.10% (357) female and 3.9% (153) male with normocytic normochromic anemia. Of the 272 with subclinical hyperthyroidism, anemia was detected in 34.19% (93) cases and types detected as follows: 19.11% (52) female and 3.3% (9) male with microcytic hypochromic anemia, and 10.66% (29) female and 1.1% (3) male with normocytic normochromic anemia. The prevalence of microcytic anemia is significantly higher within the non-euthyroid subgroup (*p* < 0.05). Mean ± SD of the different hematological parameters in thyroid dysfunction is presented in Table 5. Significantly low Hb, MCV, MCH, and HCT% were observed in the subclinical hypothyroidism and primary hypothyroidism group, particularly in females. 

### 3.4. Correlation between Thyroid Hormones and Hematological Profiles

Age was indicated to correlate with almost all of the key variables (Table 6) significantly. In both genders, age and TSH showed a positive correlation, while age and FT4 showed a negative correlation. Age showed a positive correlation with MCV, MCH, Hb, and HCT in both genders, yet a negative correlation with RBC in females and a positive correlation in males. TSH demonstrated a significant negative correlation with FT4, MCH, Hb, HCT, and RBC for both genders, while a significant positive correlation was observed for TSH with RDW in both genders. FT4 demonstrated a significant negative correlation with MCV and RDW for both genders. However, a significant positive correlation was observed for FT4 with Hb, HCT, MCHC, and RBC in both genders.

## 4. Discussion

In Saudi Arabia, the prevalence of thyroid dysfunction is constantly increasing, especially in women [25,27,28,29]. Our study aimed to assess the association of thyroid dysfunctions on the hematological profile in the Asir region of Saudi Arabia. The prevalence of thyroid dysfunction in our study was found to be 49.8%. Among them, there was 39.3% subclinical hypothyroidism, 5.3% primary hypothyroidism, 2.7% subclinical hyperthyroidism, and 2.5% primary hyperthyroidism, as shown in Figure 1. 

Iodine deficiency is the primary cause of thyroid dysfunction [30]. Altitude and thyroid dysfunction have been addressed by many researchers in Saudi Arabia, particularly in the Asir region [31,32,33,34]. Iodine, typically found in the top layer of soil, is easily washed away by erosion, particularly in high-altitude and steep-grade areas. If not preserved, topsoils at higher altitudes are more vulnerable to erosion than those at lowland areas. Crops harvested from soils deficient in iodine have low levels of iodine. Thus, the loss of iodine from the soil’s top layer may contribute to the higher prevalence of thyroid dysfunction at high altitudes than in lowland areas. The relatively high prevalence of thyroid dysfunction found in the present study may be explained by the fact that there is an endemic iodine deficiency in the Asir region (high-altitude area) of Saudi Arabia [32,33,34].

Furthermore, a clinical assessment by Al-Nuaim et al. demonstrated the correlation between the prevalence of goiter with minimum median urinary iodine concentration in the Asir region of Saudi Arabia [31]. These findings support our study regarding the high prevalence of thyroid disorder in the Asir region, especially in females. The pattern is more or less similar to studies reported in other Saudi cities [35,36].

Stratified by gender, subclinical hypothyroidism was observed in 24% (2398) females and 15.3% (1534) males. Primary hypothyroidism was observed in 2.9% (287) females and 2.4% (243) males. Subclinical hyperthyroidism was observed in 1.8% (182) females and 0.9% (90) males, and 1.4% (135) females and 1.1% (114) males were under primary hyperthyroidism. A study conducted by Rana et al. (2017) in a tertiary hospital in Riyadh, KSA, reported the high prevalence of thyroid disorders in the female Saudi adult population, and the most common was subclinical hypothyroidism [37]. In another study, Moussa et al. reported that 175 patients, 98 (71 females and 27 males) had hypothyroidism [35]. Similarly, Alrowaili et al. (2018) demonstrated that the prevalence of hypothyroidism was 25.5% and that the disease was more prevalent among females than males [38]. In general, females were found to be the majority of those with thyroid dysfunction in this study. Our observations are consistent with the previous similar studies performed in India and other countries [1,2,19,39].

The primary goal of our analysis is to determine the net effect of gender and age on the relationship between thyroid dysfunction and hematological profile. We found that females have more prevalence of thyroid disorder than males under the circumstances of thyroid dysfunction at a young age (<20 and 20–30 years), as shown in Table 3. Many researchers have reported the correlation between anemia and thyroid dysfunction, and they estimated that more than 50% of patients have blood abnormalities with thyroid dysfunction, particularly with subclinical hypothyroidism [12,13,15,16,17,40,41]. 

The most critical prevalent hematological disorder with thyroid dysfunction is anemia, and it is indicated by low Hb concentration, low MCV, low MCH, and decreased RBCs count [14,16,17,42]. Previous studies reported a low Hb level in patients with thyroid dysfunction, especially hypothyroidism [12,25,41]. Our study observed the same results, and Hb levels were significantly associated with thyroid derangement (*p* < 0.05). Similarly, Jafarzadeh reported that MCV was substantially lower in patients with thyroid dysfunction than euthyroid group; this finding supports our study [43]. Anemia was present in 35.03% (21.9% microcytic hypochromic anemia and 13.13% normocytic normochromic anemia) of the study population in this study (*p* < 0.05). Thyroid dysfunction may directly affect the process of hematopoiesis by influencing the bone marrow and erythroid precursor proliferative capacity [25,41]. Moreover, thyroid disorders may have indirect effects on erythropoiesis by inducing gene expression and secretion of erythropoietin from the kidney [9,44]. Kawa et al. studied the clinical relevance of thyroid dysfunction in human hematopoiesis. They reported that the expression of thyroid hormone receptors on human hematopoietic cells depends on the status of thyroid hormones [11]. Furthermore, the pathological condition of thyroid status significantly influences clonogenicity and induces apoptosis in CD34C-enriched hematopoietic progenitor cells [11]. Multivariate analysis was performed and showed that the most affected hematological parameters by thyroid disorders were the Hb, MCV, MCH, HCT, and RBC, respectively (*p* < 0.05). However, RDW in males does not show any significant correlation with thyroid disorders (*p* < 0.05), which is in agreement with other studies reported earlier [9,12,14].

Our research has some limitations; for instance, weight and body mass index are not included in our study. Additionally, this is a cross-sectional study, and therefore, the cause-and-effect relationship cannot be differentiated. Additionally, no serum ferritin or serum iron data were available since iron deficiency is frequently linked to subclinical hypothyroidism, particularly in females. Furthermore, follow-up patients were not included in this study, and thyroid data are provided once. One other limitation is that the results cannot be applied to other populations, but they are limited to the particular region understudy in this investigation. Despite the limitations of this population-based study, thyroid disorder is highly prevalent in the Asir region of Saudi Arabia. The majority of our study population with subclinical hypothyroidism were young and predominantly females.

## 5. Conclusions

Our study found that 49.8% of the study population had a thyroid disorder. There was 39.3% subclinical hypothyroidism, 5.3% primary hypothyroidism, 2.7% subclinical hyperthyroidism, and 2.5% primary hyperthyroidism. Most of the thyroid disorder cases were females. Furthermore, our research found that thyroid dysfunction, especially hypothyroidism, affects most hematological parameters, with these effects being more prominent in females. Microcytic hypochromic anemia and normocytic normochromic anemia were the most prevalent types associated with thyroid dysfunction. A significant relation was observed between the TSH, FT4, age, sex, and different hematological parameters (*p* < 0.05). Therefore, we recommend a national strategy to control the risk of thyroid abnormalities in our country, health education, and awareness to the general population about the nature of the thyroid disorder and associated risk factors. Additionally, a control program is needed to ensure the exclusive availability of iodized salt throughout Saudi Arabia, particularly in the southern provinces. A nationwide salt iodination program must be implemented to address thyroid disorder in the affected area and avoid iodine deficiency in other parts of the country. Furthermore, people should be encouraged to check-up for thyroid function tests periodically.

## Figures and Tables

**Table 1:** Gender and age subgroup of the study population.

Age Range (Years)	Gender	
	FEMALE% (*n* = 6146)	MALE% (*n* = 3831)
Below 20	4.7% (290)	6.0% (229)
20–30	14.5% (895)	9.8% (376)
31–40	31.6% (1949)	26.9% (1030)
41–50	20.4% (1259)	22.4% (861)
Above 50	28.7% (1767)	34.9% (1336)

**Table 2:** Characteristics of the study population stratified by gender.

Parameters	Female		Male		*p* *
	Mean ± SD	Median ± IQR	Mean ± SD	Median ± IQR	
TSH (μIU/mL)	4.32 ± 6.7	2.86 ± 4.1	4.22 ± 5.8	2.90 ± 4.1	0.668
FT4 (ng/dL)	0.99 ± 0.17	0.98 ± 0.2	1.01 ± 1.3	0.99 ± 0.2	0.066
Hb (g/dL)	12.84 ± 2.1	13.90 ± 2.3	14.75 ± 2.1	15.0 ± 2.6	0.039
HCT (%)	40.73 ± 5.1	40.80 ± 6.0	42.75 ± 5.7	43.40 ± 6.8	0.000
MCV (fL)	79.7 ± 9.7	81.60 ± 7.2	80.27 ± 8.4	81.35 ± 5.9	0.000
MCH (Pg)	26.92 ± 4.0	27.80 ± 3.6	27.42 ± 3.6	28.10 ± 3.1	0.000
MCHC (g/dL)	33.95 ± 1.6	34.10 ± 1.9	34.33 ± 1.5	34.50 ± 1.9	0.000
RDW (%)	14.38 ± 2.1	13.80 ± 1.9	14.09 ± 1.8	13.60 ± 1.6	0.000
RBC (10^12^/L)	4.82 ± 1.3	5.01 ± 0.8	5.17 ± 1.1	5.34 ± 0.8	0.000

* Analyzed by Mann–Whitney U-test.

**Table 3:** Prevalence of thyroid dysfunction on different genders stratified by age subgroup.

Thyroid Dysfunction	Prevalence in Different Age Sub-Group (Years)						
	Below 20	20–30	31–40	41–50	>50	Total	χ^2^
**Female**							102.18 ^a^ (*p =* 0.000) ^a^ 105.32 ^b^ (*p* = 0.000) ^b^
Euthyroidism	53.1% (154)	51.6% (462)	52.6% (1025)	50.6% (637)	49.8% (880)	31.6% (3158)	
Primary Hypothyroidism	1.7% (5)	3% (27)	3% (59)	6.5% (81)	6.5% (115)	2.9% (287)	
Primary Hyperthyroidism	0.3% (1)	1.7% (15)	1.6% (32)	1.8% (22)	3.7% (65)	1.4% (135)	
Subclinical Hypothyroidism	44.8% (130)	41.2% (369)	40.6% (789)	37.6% (473)	36.0% (637)	24.0% (2398)	
Subclinical Hyperthyroidism	0.0% (0)	2.5% (22)	2.3% (44)	3.7% (46)	4.0% (70)	1.8% (182)	
**Male**							59.16 ^a^ (*p =* 0.000) ^a^ 69.67 ^b^ (*p =* 0.000) ^b^
Euthyroidism	51.1% (117)	51.1% (192)	49% (505)	49.5% (427)	46.4% (620)	18.6% (1861)	
Primary Hypothyroidism	0% (0.0)	4.5% (17)	4.8% (49)	7% (60)	8.8% (117)	2.4% (243)	
Primary Hyperthyroidism	0.4% (1)	2.7% (10)	2.2% (23)	3.6% (31)	3.7% (49)	1.1% (114)	
Subclinical Hypothyroidism	48% (110)	40.4% (152)	41.6% (428)	37.3% (321)	38.4% (513)	15.3% (1524)	
Subclinical Hyperthyroidism	0.4% (1)	1.3% (5)	2.4% (25)	2.6% (22)	2.8% (37)	0.9% (90)	

^a^ Pearson chi-square, ^b^ Fisher’s exact test.

**Table 4:** Prevalence and types of thyroid dysfunction and anemia.

Anemia		Euthyroidism (*n* = 5019)	Primary Hypothyroidism (*n* = 530)	Primary Hyperthyroidism (*n* = 249)	Subclinical Hypothyroidism (*n* = 3922)	Subclinical Hyperthyroidism (*n* = 272)	χ^2^
Non-anemic (*n* = 6491)	Female (*n* = 3645)	30.6% (1987)	2.1% (137)	1.0% (66)	20.9% (1354)	1.6% (101)	86.82 ^a^ (*p* = 0.000) ^a^ 86.39 ^a^ (*p* = 0.000) ^b^
	Male (*n* = 2846)	20.6% (1340)	3.4 (221)	1.6% (106)	17.0% (1101)	1.2% (78)	
Microcytic hypochromic (*n* = 2189)	Female (*n* = 1574)	32.4% (709)	4.2% (92)	1.6% (34)	31.4% (687)	2.4% (52)	28.38 ^a^ (*p* = 0.000) ^a^ 30.81 ^b^ (*p* = 0.000) ^b^
	Male (*n* = 615)	14.6% (319)	0.5% (12)	0.2% (5)	12.3% (270)	0.4% (9)	
Normocytic normochromic (*n* = 1312)	Female (*n* = 941)	35.2% (462)	4.4% (58)	2.7% (35)	27.2% (357)	2.2% (29)	21.84 ^a^ (*p* = 0.000) 23.87 ^b^ (*p* = 0.000) ^b^
	Male (*n* = 371)	15.4% (202)	0.8% (10)	0.2% (3)	11.7% (153)	0.2% (3)	

Analyzed by ^a^ Pearson chi-square, ^b^ Fisher’s exact test.

**Table 5:** Mean ± SD of hematological parameters in thyroid dysfunction group stratified by gender.

Thyroid Dysfunction	Hb (g/dL)	MCV (fL)	MCH (pg/cell)	HCT (%)	RBC (×10^6^)	MCHC (g/dL)	RDW (%)
**Female**							
Euthyroidism	14.02 ± 2.01	81.94 ± 9.5	27.06 ± 4.01	41.05 ± 5.11	4.82 ± 1.34	34.08 ± 1.51	14.28 ± 1.97
Primary Hypothyroidism	11.22 ± 1.93	79.48 ± 11.2	26.67 ± 4.15	39.86 ± 5.60	4.73 ± 0.94	33.07 ± 1.73	14.61 ± 2.22
Primary Hyperthyroidism	13.48 ± 1.32	80.66 ± 6.86	27.36 ± 2.43	40.30 ± 3.52	4.96 ± 0.64	33.43 ± 1.32	14.22 ± 1.76
Subclinical Hypothyroidism	11.83 ± 2.14	79.17 ± 9.92	26.76 ± 4.11	39.48 ± 5.02	4.78 ± 1.34	33.96 ± 1.62	14.48 ± 2.03
Subclinical Hyperthyroidism	13.55 ± 1.76	79.74 ± 7.21	26.93 ± 2.92	40.17 ± 4.24	5.03 ± 0.52	33.65 ± 1.56	14.49 ± 1.87
*p* value	0.000	0.002	0.035	0.000	0.080	0.000	0.001
**Male**							
Euthyroidism	14.65 ± 2.12	82.10 ± 9.10	27.35 ± 3.8	42.40 ± 5.82	5.14 ± 1.12	34.34 ± 1.51	14.08 ± 1.79
Primary Hypothyroidism	12.72 ± 1.59	79.10 ± 7.50	28.33 ± 2.9	40.90 ± 6.62	4.91 ± 0.92	34.41 ± 1.56	13.82 ± 1.54
Primary Hyperthyroidism	15.60 ± 1.34	80.10 ± 5.10	27.90 ± 2.1	44.90 ± 7.09	5.51 ± 0.80	33.98 ± 1.44	14.24 ± 2.09
Subclinical Hypothyroidism	12.62 ± 2.14	79.10 ± 7.90	27.35 ± 3.6	40.62 ± 5.11	4.81 ± 1.15	34.34 ± 1.50	14.13 ± 1.75
Subclinical Hyperthyroidism	15.23 ± 1.68	80.01 ± 5.91	27.40 ± 2.31	43.25 ± 7.82	5.48 ± 0.79	34.22 ± 1.35	14.21 ± 1.85
*p* value	0.000	0.002	0.001	0.000	0.000	0.107	0.111

**Table 6:** A correlation coefficient of key variables based on gender.

Key Variables	Female			Male		
	TSH	FT4	Age	TSH	FT4	Age
Age	0.40 **	−0.15 *	-	0.12 *	−0.76 **	-
TSH	-	−0.161 **	0.40 **	-	−0.165 **	0.12
FT4	−0.161 **	-	−0.15	−0.165 **	-	−0.76 **
MCV	−0.017	−0.045 **	0.085 **	−0.018	−0.048 **	0.090 **
MCH	−0.022	0.012	0.071 **	−0.012	−0.016	0.077 **
Hb	−0.048 **	0.120 **	0.004	−0.075 **	0.098 **	0.056 **
HCT	−0.050 **	0.099 **	0.019	−0.067 **	0.082 **	0.056 **
MCHC	−0.016	0.111 **	−0.035 **	−0.017	0.076 **	−0.017
RDW	0.041 **	−0.058 **	−0.001	0.045 **	−0.041 *	−0.001
RBC	−0.053 **	0.130 **	−0.042 **	−0.074 **	0.139 **	0.014

* *p* < 0.05, ** *p* < 0.01.

## Supplementary Materials

The following are available online at https://www.mdpi.com/article/10.3390/clinpract11030065/s1, Table S1: Overall prevalence of thyroid disorder in study population, stratified by age-subgroup.
