# Supplementary material for: Prevalence and Characteristics of Thyroid Abnormalities and Its Association with Anemia in ASIR Region of Saudi Arabia: A Cross-Sectional Study
Source: Clin Pract. 2021 Aug 6;11(3):494–504. doi: 10.3390/clinpract11030065 (PMC8395449; doi:10.3390/clinpract11030065)
Supplement: Supplementary file 1 [file clinpract-11-00065-s001.zip › clinpract-1255364-supplementary.pdf]

Table S1. Overall prevalence of thyroid disorder in study population, stratified by age-subgroup.

		Thyroid Function					
		**Euthyroidism**	**Primary hypothyroidism**	**Primary Hyperthyroidism**	**Subclinical Hypothyroidism**	**Subclinical Hyperthyroidism**	**Total**
**Age range**	Below 20	(2.7%) 271	(0.1%) 5	0.02% (2)	(2.4%) 240	(0.01%) 1	519
	20-30	(6.5%) 654	(0.4%) 44	(0.3%) 25	(5.2%) 521	(0.3%) 27	1271
	31-40	(15.3%) 1530	(1.1%) 108	(0.6%) 55	(12.2%) 1217	(0.7%) 69	2979
	41-50	(10.6%)1064	(1.4%) 141	(0.5%) 53	(7.9%) 794	(0.7%) 68	2120
	More than 50	(15%) 1500	(2.3%) 232	(1.1%) 114	(11.5%) 1150	(1.1%) 107	3103
Total		(50.2%) 5019	(5.3%) 530	(2.5%) 249	(39.3%) 3922	(2.7%) 272	9992
